# Design of Optimized Hypoxia-Activated Prodrugs Using Pharmacokinetic/Pharmacodynamic Modeling

**DOI:** 10.3389/fonc.2013.00314

**Published:** 2013-12-27

**Authors:** Annika Foehrenbacher, Timothy W. Secomb, William R. Wilson, Kevin O. Hicks

**Affiliations:** ^1^Auckland Cancer Society Research Centre, The University of Auckland, Auckland, New Zealand; ^2^Department of Physiology, University of Arizona, Tucson, AZ, USA

**Keywords:** tumor hypoxia, hypoxia-activated prodrugs, bystander effect, extravascular drug transport, pharmacokinetic/pharmacodynamic modeling, rational drug design, tirapazamine, PR-104

## Abstract

Hypoxia contributes to resistance of tumors to some cytotoxic drugs and to radiotherapy, but can in principle be exploited with hypoxia-activated prodrugs (HAP). HAP in clinical development fall into two broad groups. Class I HAP (like the benzotriazine *N*-oxides tirapazamine and SN30000), are activated under relatively mild hypoxia. In contrast, Class II HAP (such as the nitro compounds PR-104A or TH-302) are maximally activated only under extreme hypoxia, but their active metabolites (effectors) diffuse to cells at intermediate O_2_ and thus also eliminate moderately hypoxic cells. Here, we use a spatially resolved pharmacokinetic/pharmacodynamic (SR-PK/PD) model to compare these two strategies and to identify the features required in an optimal Class II HAP. The model uses a Green’s function approach to calculate spatial and longitudinal gradients of O_2_, prodrug, and effector concentrations, and resulting killing in a digitized 3D tumor microregion to estimate activity as monotherapy and in combination with radiotherapy. An analogous model for a normal tissue with mild hypoxia and short intervessel distances (based on a cremaster muscle microvessel network) was used to estimate tumor selectivity of cell killing. This showed that Class II HAP offer advantages over Class I including higher tumor selectivity and greater freedom to vary prodrug diffusibility and rate of metabolic activation. The model suggests that the largest gains in class II HAP antitumor activity could be realized by optimizing effector stability and prodrug activation rates. We also use the model to show that diffusion of effector into blood vessels is unlikely to materially increase systemic exposure for realistic tumor burdens and effector clearances. However, we show that the tumor selectivity achievable by hypoxia-dependent prodrug activation alone is limited if dose-limiting normal tissues are even mildly hypoxic.

## Introduction

Prodrugs that are enzymatically converted to active metabolites (effectors) within tumors are of interest for selective cancer therapy. Three different strategies have been explored for intra-tumor prodrug activation: (1) xenobiotic metabolizing enzymes that are highly expressed in specific tumors (1, 2); (2) exogenous enzymes targeted to tumors using antibody (3), viral (4), or bacterial (5) vectors; (3) endogenous enzymes that reduce prodrugs only under the hypoxic conditions that prevail in tumors. Hypoxia-activated prodrugs (HAP), which provide this third strategy, not only exploit a generic feature that differentiates tumors from most normal tissues but also potentially overcome the resistance of hypoxic cells to radiotherapy and many chemotherapy drugs (6–15). Several HAP have been evaluated clinically (16–19), including ongoing studies with the nitro compounds TH-302 (20, 21), PR-104A [(22–25); administered as the corresponding phosphate ester PR-104] and PR-610 (26). However, many questions remain as to how HAP should be designed for optimal anticancer activity.

**Figure 1:**
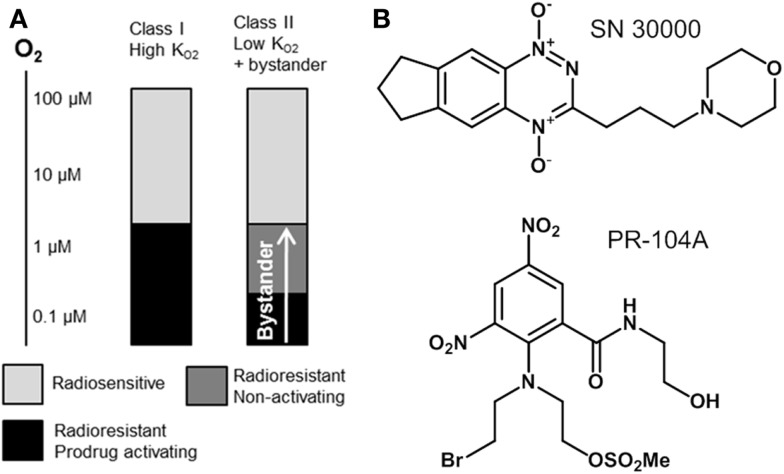
**Hypoxia-activated prodrug strategies**. **(A)** Schematic representation of complementary cell killing achieved by radiation in combination with a high-$K_{O_{2}}$ HAP and a low-$K_{O_{2}}$ HAP that generates active metabolites that diffuse out of prodrug-activating zones to kill neighboring cells (bystander). **(B)** Examples of Class I (SN30000) and Class II (PR-104A) HAP are shown.

In the present study we utilize pharmacokinetic/pharmacodynamic (PK/PD) models to explore relationships between the reaction/diffusion properties of HAP in the tumor microenvironment and their antitumor activity and selectivity. In this context it is useful to distinguish two broad classes of HAP with different PK/PD features (Figure 1). Class I HAP are activated by reduction under relatively mild hypoxia to generate a reactive cytotoxin, which is restricted to the cell in which it is formed. This class is typified by the benzotriazine *N*-oxides tirapazamine and SN30000 that undergo 1-electron reduction to short-lived cytotoxic radicals (27, 28), which appear not to affect adjoining cells in three-dimensional (3D) cell cultures (29). The O_2_ concentration for 50% inhibition of cytotoxicity ($K_{O_{2}}$) in stirred single cell suspensions is approximately 1 μM for both tirapazamine (30, 31) and SN30000 (32). Class II HAP (typified by nitro compounds such as PR-104A and TH-302) require more severe hypoxia for 1-electron reduction, but the resulting radicals are further reduced to a relatively stable effector that can diffuse from the cell of origin (29, 33–35); this diffusion of effector to nearby cells (which may not themselves be capable of prodrug activation) is here referred to as a bystander effect. In the case of PR-104A, a $K_{O_{2}}$ value of ~0.1 μM has been estimated in stirred single cell suspensions (31), and activation of TH-302 also appears to require severe hypoxia (34) as is the case for other nitro compounds (36–38). Given that half-maximal radiosensitization of tumor cells by oxygen requires concentrations of ~4 μM (39, 40), the ability of Class II HAP to overcome radioresistance may depend on bystander effects.

We have suggested (41) that Class II HAP may be preferable to Class I because activation will be confined to the extreme hypoxia found in tumors, thus minimizing toxicity to normal tissues with mild, physiological hypoxia [e.g., retina (42), liver (43–45), esophagus (46), skin (47, 48), and possibly the bone marrow stem cell niche (49) although the oxygenation status of the latter is controversial (50)]. The bystander effect from Class II HAP may contribute to the reported monotherapy activity of PR-104 (33, 51, 52) and TH-302 (53) in preclinical models. However, it is not known under what conditions this theoretical advantage for Class II HAP might be realized, or how this activity might be optimized by prodrug design, or whether diffusion of active metabolites into the tumor microvasculature might contribute to systemic toxicity if this process is too efficient.

**Figure 2:**
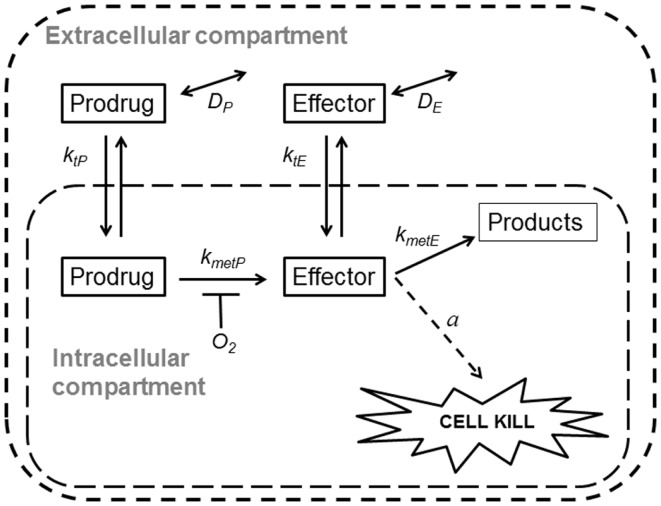
**Schematic representation of a generalized HAP PK/PD model**. Transfer of prodrug and effector between the extracellular and the intracellular compartment is defined by rate constant *k*_t_*_N_*, where *N* refers to each compound. In the extracellular compartment compounds can diffuse as defined by their diffusion coefficients *D_N_* (double-headed arrows). Prodrug activation (defined by *k*_met_*_P_*) is restricted to the intracellular compartment and is O_2_-dependent (see Eq. 3). For an effector that confers cytotoxicity by way of reacting irreversibly with its target (case 1, e.g., DNA-alkylating agents) the model assumes that potency *a* scales with reactivity of the effector (*k*_met_*_E_*), while in case 2 (e.g., a reversible inhibitor) *a* is independent of*k*_met_*_E_*.

Spatially resolved pharmacokinetic/pharmacodynamic (SR-PK/PD) models provide tools for addressing these questions. These models can be used to describe concentration gradients of oxygen, HAP, and their effectors in tumors, using mapped microvascular networks, and to calculate resulting reproductive cell death (clonogenic cell killing). These models include the effects of heterogeneity in inter-capillary distances, vessel diameters, blood flow rates, and vessel oxygen and drug concentrations. We have validated an SR-PK/PD model for Class I HAP by showing that it predicts activity of tirapazamine analogs combined with radiotherapy in human tumor xenografts (32, 54, 55). This modeling clearly demonstrated the need to optimize rates of reductive metabolism such that penetration into hypoxic regions is not compromised by excessive consumption of the prodrug. Recently, we have also reported an SR-PK/PD model for the Class II HAP, PR-104, and used this to estimate that 30–50% of its activity in HCT116 and SiHa xenografts is due to bystander effects both as monotherapy and combined with radiation (35). Here, we use a generalized SR-PK/PD model, in which a HAP is metabolized by an oxygen-inhibited process to a single effector (Figure 2), to ask under what conditions Class II HAP might provide greater tumor activity and selectivity than Class I HAP, and to identify the prodrug features required for optimal antitumor activity. This generalized HAP model makes explicit the diffusion of both the prodrug and effector in the extracellular (interstitial) compartment. We consider two types of effector, which elicit cytotoxicity via irreversible reaction with a target (Case 1, as for an alkylating agent) or by reversible binding to its target (Case 2, as for a receptor ligand).

## Methods

The generalized SR-PK/PD model calculates steady-state concentrations of oxygen, HAP, and effector as well as resulting cell killing in digitized 3D tissue microregions using Green’s function methods (35, 54, 56). We used two different tissue microregions that were derived by mapping microvascular anatomy as well as direction and velocity of blood flow in a rat cremaster muscle (56) (“normal” network) and a subcutaneous FaDu tumor xenograft (57) (“tumor” network). The blood vessels are represented by cylindrical segments and vessel walls are treated as part of the tissue space. The model was implemented using a customized version of the Green’s function method written in Visual C++ (Microsoft Visual Studio 2010 Express) (35, 58).

### Calculation of oxygenation

Convective transport of oxygen along vessel segments and diffusion into the surrounding tissue (represented as homogeneous medium) was calculated based on previous estimates for blood flow in the networks, blood O_2_ content, tissue diffusion, and consumption of O_2_ (35, 56). All O_2_ transport parameters are summarized in Table 1.

**Table 1:** **O_2_ transport parameters used to model oxygenation in the FaDu tumor and the cremaster muscle microregions**.

Parameter	Unit	Value for		Description
		FaDu	Cremaster	
*V*	mm^3^	0.12	0.091	Volume of the mapped region
*Q*	nl/min	40.0	75.0	Total blood inflow to region = sum of blood flow in all feeding arterioles
*Q/V*	nl/min/mm^3^	333	824	
*p*O_2_	mm Hg	40.0	50.0	O_2_ partial pressure in inflowing vessels
*P*_50_	mm Hg	38.0	38.0	O_2_ partial pressure for half saturation of hemoglobin
*N*		3.0	3.0	Hill coefficient for O_2_ binding to hemoglobin
*C*_0_	cm^3^ O_2_/mm Hg	0.5	0.5	O_2_ binding capacity of red blood cells
*H*_D_		0.4	0.4	Discharge hematocrit
α	cm^3^ O_2_/cm^3^/mm Hg	3.1 × 10^−5^	3.1 × 10^−5^	O_2_ solubility
$\alpha D_{O_{2}}$	cm^3^ O_2_/cm/s/mm Hg	4.2 × 10^−10^	9.4 × 10^−10^	Krogh diffusion coefficient for O_2_ diffusion
$D_{O_{2}}$	cm^2^/s	1.4 × 10^−5^	3.0 × 10^−5^	O_2_ diffusion coefficient
*M*_0_	cm^3^ O_2_/cm^3^/s	5 × 10^−4^	9.5 × 10^−4^	Rate of O_2_ consumption when supply is not limiting
*K_m_*_O2_	mm Hg	1.0	1.0	Michaelis constant for O_2_ consumption

**Figure 3:**
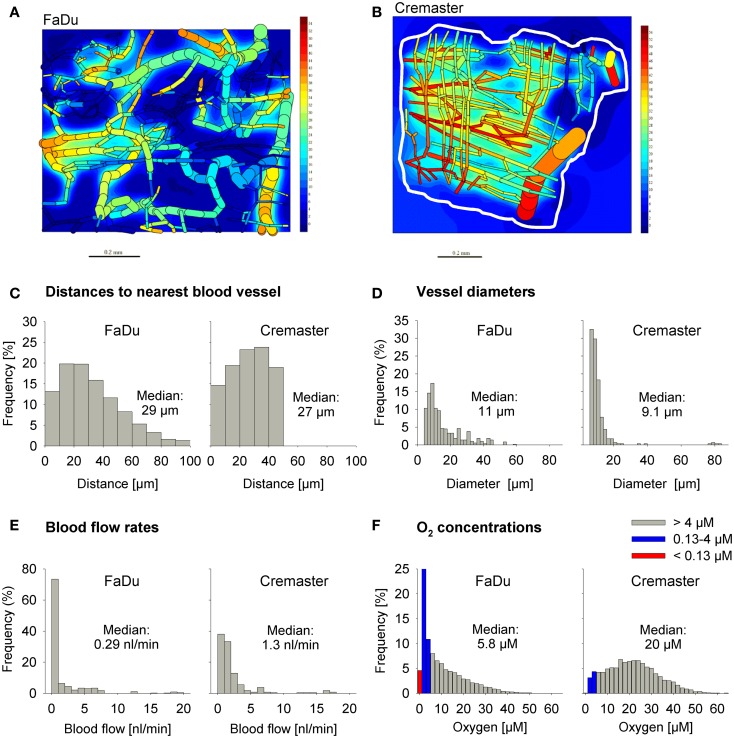
**Virtual tissue microregions used for SR-PK/PD modeling**. Oxygenation in the FaDu tumor microregion [990 μm × 810 μm × 150 μm; **(A)**] and the cremaster muscle region [1100 μm × 1000 μm × 240 μm; **(B)**] was modeled using the O_2_ transport parameters reported in Table 1. **(A,B)** Show contour plots of O_2_ (in mm Hg) in a mid-plane section of the microregions, superimposed with the whole microvascular network projected into one plane. The dimensions of the mapped cremaster muscle network are not cuboidal and O_2_ concentrations were only computed for tissue points <50 μm away from any vessel (approximately indicated by a white line). **(C–F)** Show frequency histograms of distances to the nearest blood vessel **(C)**, vessel diameters **(D)**, blood flow rates **(E)**, and O_2_ concentrations **(F)**. The red column in **(F)** represents the fraction at 0–0.13 μM O_2_ (i.e., below the $K_{O_{2}}$ value for a representative Type II HAP).

*Parameters were as reported previously for the FaDu (35) and the cremaster muscle microregion (56) except that *M*_0_ in the cremaster region was adjusted to achieve the required hypoxic fraction. The chosen *M*_0_ value is intermediate between the values previously used to represent mild and moderate levels of muscle activity (56). These O_2_ transport parameters gave hypoxic fractions and median *p*O_2_ values (Figure 3) in the range measured for tumors and normal tissues in humans using O_2_ electrode histography (45)*.

### Calculation of pharmacokinetics

Inflow of prodrug to the microvascular networks was defined by its unbound plasma level, using area under the concentration-time curve (AUC) as a time-independent exposure variable compatible with Green’s function formalism. Vessel walls were modeled as offering negligible resistance to radial flux by setting the intravascular resistance constant *K* (56) to a low value (0.1 s/μm). Extravascular transport was calculated using a (pro)drug transport model (Figure 2) assuming that the tissue consists of two homogeneous (extracellular and intracellular) compartments, with activation of prodrug restricted to the intracellular compartment and diffusion confined to the extracellular compartment. Concentrations in the two compartments were calculated as follows:

(1) $$\varphi_{\text{e}}\frac{\partial {C_{e}}_{N}}{\mathrm{\partial t}}=D_{N}\nabla^{2}C_{\text{e}N}-\varphi_{i}k_{tN}\left(C_{\text{e}N}-C_{\text{i}N}\right)$$

(2) $$\varphi_{i}\frac{\partial C_{\text{i}N}}{\mathrm{\partial t}}=\varphi_{i}k_{tN}\left(C_{\text{e}N}-C_{\text{i}N}\right)-\varphi_{i}k_{\text{met}N}C_{\text{i}N}+r_{N}$$

*C*_e_ and *C*_i_ are the extracellular and intracellular concentrations, respectively, of prodrug or effector (denoted by *N* = *P* or *E*), φ_i_ and φ_e_ are the intra- and extracellular volume fractions with φ_e_ = 1 − φ_i_, *k*_t_*_N_* are first order rate constants for cell transfer between the extracellular and intracellular compartments, *D_N_* is the effective diffusion coefficient of compound *N*, ▽^2^ is the Laplacian operator, *k*_met_*_N_* is the rate constant for drug metabolism and *r* is the rate of metabolic production with *r_P_* = 0 and rate of effector production equal to the rate of loss of prodrug: *r_E_* = φ*_i_k*_met_*_P_C*_i_*_P_*. The rate constant for prodrug activation is O_2_-dependent and is given by:

(3) $$k_{\text{met}P}=\left(\frac{K_{O_{2}}}{K_{O_{2}}+[{\text{O}}_{2}]}\right)k_{\text{met}P,\max}$$

where *k*_met_*_P_*_,max_ and *k*_met_*_P_* are the prodrug activation rate constants under anoxia and at the O_2_ concentration [O_2_], respectively, and $K_{O_{2}}$ is the O_2_ concentration for half-maximum prodrug activation.

### Calculation of cell killing

Survival probability (SP) at each point of the tumor microregion was calculated using the PK/PD relationship:

(4) $$-\log\;Survival\;Probability\;\left(SP\right)=aAUC_{iE}$$

where AUC_i_*_E_* is the intracellular exposure (area under the intracellular concentration-time curve) to the effector and *a* is a proportionality constant (potency) equal to the reciprocal of the effector AUC to produce a SP of 0.1 as measured by clonogenic cell survival.

We distinguish two cases: case 1, where potency *a* scales with *k*_met_*_E_*, broadly representing irreversible inhibitors such as DNA-alkylating agents for which an increase in reactivity has similar effects on reaction with target and non-target nucleophiles (59, 60) and case 2, where *a* is independent of *k*_met_*_E_* (broadly representing reversible inhibitors). Case 1, where cell killing is proportional to the reactivity of the effector is used throughout this study unless noted otherwise.

To estimate cell SP after radiation treatment, the linear-quadratic model was used as previously (35, 54). The SP from both radiation and prodrug at each point of the tumor microregion was calculated from the sum of log (SP) due to drug and radiation alone. Log cell kill was then calculated as

(5) logcellkill=−log(AverageSP)

where the SP is averaged over the whole tumor microregion. Prodrug-induced cell kill in addition to radiation was calculated as the difference between overall log cell kill by prodrug + radiation and log cell kill by radiation alone.

### Estimation of net transport across the tumor-plasma boundary

The SR-PK/PD model calculates the transport of prodrug and effector across the walls of the 582 vessel segments in the FaDu tumor microvascular network. Summing the 582 values for each compound gives the net transport across the tumor-plasma boundary (in picomoles). This was normalized to the volume of the FaDu tumor microregion.

### Compounds

PR-104H (33) and PR-104M (61) were prepared from PR-104A as described and stored in acetonitrile at −80°C. Tetradeuterated internal standards of PR-104H (PR-104H-d4) and PR-104M (PR-104M-d4) were synthesized using the same methods as for the non-deuterated compounds. PR-104H and PR-104H-d4 had a purity of >90% by HPLC while PR-104M and PR-104M-d4 had a purity of 86 and 84%, respectively.

### Pharmacokinetic studies in mice

All animal studies were approved by the University of Auckland Animal Ethics Committee. Male NIH-III nude mice (NIH-Lyst^bg^Foxn1^nu^Btk^xid^; 18–20 g body weight), derived from breeding mice purchased from Charles River Laboratories (Wilmington, MA, USA), were dosed i.v. with PR-104H. The dosing solution was prepared fresh for each mouse by dilution of a stock solution of PR-104H in acetonitrile with sterile saline (to <20% acetonitrile) within 3 min of dosing to minimize loss of PR-104H in aqueous solution. At the dose volume of 0.005 ml/g the acetonitrile dose was 755 mg/kg and the vehicle alone did not cause signs of toxicity over the duration of the experiment. Blood was collected by cardiac puncture up to 3 min after cervical dislocation as in (62). Plasma was prepared as described (63) and mixed with three volumes of cold acidified methanol (methanol:ammonium acetate:acetic acid 1000:3.5:0.2, v/w/v) ~6 min after cardiac puncture. Liver tissue was dissected within 3 min of blood sampling, snap-frozen in liquid nitrogen, and stored at −80°C along with plasma samples. Frozen tissue was pulverized at liquid nitrogen temperature using a BioPulverizer™ (BioSpec Products, USA), transferred to tared tubes, weighed, vortex mixed for 30 s with an equal volume of cold phosphate buffered saline and six volumes of cold acidified methanol, and stored at −80°C. For LC-MS/MS analysis [as reported in Ref. (35)], tissue and plasma extracts were centrifuged (13,000 × *g*, 4°C, 10 min) and supernatants were mixed 1:1 with cold water containing 1 μM PR-104H-d4 and 1 μM PR-104M-d4.

## Results

In order to identify optimal properties of HAP, we developed a SR-PK/PD model that captures the key features of both Class I and Class II HAP (Figure 2). This model is a generalized form of our SR-PK/PD model for PR-104 (35), which, unlike earlier SR-PK/PD models (31, 32, 54) accommodates diffusion and reaction of the effector and explicitly considers intra- and extracellular compartments in order to represent the plasma membrane barrier to prodrug uptake and effector efflux. Our generalized model assumes that the effector can be further converted to inactive products within the cell (defined by rate constant *k*_met_*_E_*). The model incorporates only one effector (unlike the two alkylating metabolites from PR-104A) and represents an idealized HAP that is not a substrate for O_2_-insensitive two-electron-reduction, which is an off-target activation mechanism of some HAP including PR-104A (51). We also ignore any systemic generation of effector outside the modeled tissue region, such as from hepatic metabolism.

### Tissue microregions used for SR-PK/PD modeling

We used a digitized 3D tumor microregion (Figure 3A) that was derived by mapping a region of a subcutaneous FaDu tumor xenograft grown in a mouse dorsal window chamber (57). To assess HAP activation under conditions of physiological hypoxia we also used a 3D region of a rat cremaster muscle (56) as an example of a normal tissue with a well-organized and efficient microvascular network (Figure 3B). While the maximum distance to the nearest blood vessel is 50 μm in the cremaster muscle region, 20% of tissue points in the FaDu tumor region are situated further from blood vessels (50–100 μm; Figure 3C). The FaDu tumor region also showed a higher heterogeneity in vessel diameters (Figure 3D), and a lower median blood flow rate (Figure 3E) at the values chosen for total blood inflow into the regions (*Q*; Table 1). *Q* per tissue volume was 2.5-fold lower in the FaDu tumor than in the cremaster muscle microregion, which is realistic for a low-perfused hypoxic tumor area. The value for the tumor microregion falls within the range of mean blood flow values measured in human tumors, which show high variability with values that can be lower, similar, or higher than in the tissue of origin (64, 65). A radiobiological hypoxic fraction (<4 μM O_2_) of 43% was achieved in the FaDu tumor microregion (Figure 3F) by using O_2_ transport parameters previously chosen to model the hypoxic fraction of tumor xenografts (35) (Table 1). The O_2_ transport parameters for the cremaster muscle region were as reported (56), with the oxygen consumption rate adjusted to achieve a radiobiological hypoxic fraction of 7.5% (Figure 3F) that is consistent with moderate hypoxia as measured in some normal human tissues using O_2_ electrode histography (45). However, the muscle region lacks the severely hypoxic cells (defined as <0.13 μM O_2_, i.e., below the $K_{O_{2}}$ value for PR-104A) that comprise 5.0% of the FaDu microregion (Figure 3F).

### Modulation of kinetics of prodrug activation

Our previous SR-PK/PD model for Class I HAP showed that high prodrug activation rates limit killing of hypoxic cells by impairing tissue penetration of the prodrug (54). To investigate whether this also applies to HAP with bystander effects and/or activation restricted to more severe hypoxia (low $K_{O_{2}}$), we modulated the prodrug activation rate constant for anoxia (*k*_met*P*,max_) under these conditions. For simulations without a bystander effect, *k*_t_*_E_* was set to 0, which traps effector within the intracellular compartment.

**Figure 4:**
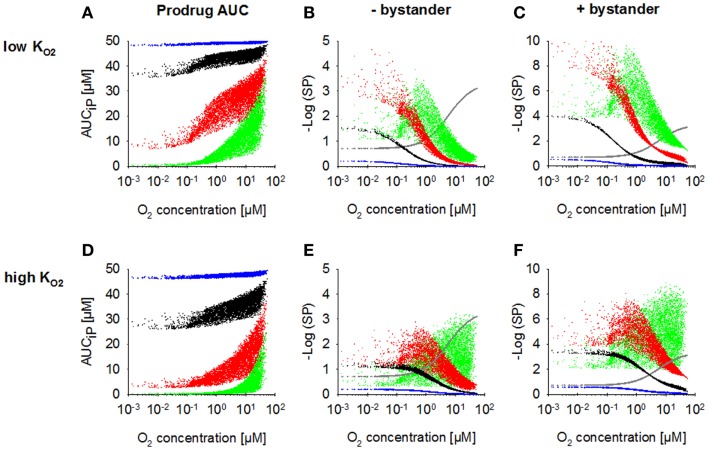
**Dependence of PK/PD in the FaDu tumor microregion on prodrug activation rate constant *k*_met*P*,max_**. SR-PK/PD model simulations for HAP with $K_{O_{2}}$ = 0.13 μM **(A–C)** and a 10-fold higher $K_{O_{2}}$ of 1.3 μM **(D–F)** are shown as a function of O_2_ concentration using a *k*_met*P*,max_ of 0.001 s^−1^ (blue), 0.01 s^−1^ (black), 0.1 s^−1^ (red), or 1 s^−1^ (green). **(A,D)** Intracellular prodrug AUC (AUC_i_*_P_*). **(B,E)** Survival probability after HAP exposure or 15 Gy radiation (gray) in the absence of bystander effects. **(C,F)** Survival probability with bystander effects. For no-bystander simulations, the rate constant for membrane transfer of the effector *k*_t_*_E_* was set to 0 and effector potency was set to a lower value of 0.0414 μM^−1^h^−1^, to achieve similar average tumor cell kill as in the bystander case. All remaining parameters were as reported in Table 2.

Increasing *k*_met*P*,max_ resulted in a decrease of prodrug penetration to the most hypoxic regions, an effect that was less pronounced with a low $K_{O_{2}}$ (Figure 4A) than with a high $K_{O_{2}}$ value (Figure 4D), as expected. Accordingly, killing in the most hypoxic regions (<0.13 μM O_2_) was higher and less affected by an increase in *k*_met*P*,max_ for low-$K_{O_{2}}$ HAP (Figures 4B,E; without bystander effects). The observed shift of cell killing to higher O_2_ concentrations with increasing *k*_met*P*,max_ reflects increased prodrug activation at intermediate O_2_ concentrations. In case of low-$K_{O_{2}}$ HAP this improved complementation of radiation-induced killing (Figure 4B), but in case of high-$K_{O_{2}}$ HAP it resulted in a loss of hypoxic selectivity at a 100-fold higher *k*_met*P*,max_ (Figure 4E). If the effector was allowed to diffuse through the plasma membrane (i.e., the bystander model), killing was increased across the whole tumor microregion, and killing <0.13 μM was less affected by decreasing prodrug penetration with increasing *k*_met*P*,max_ (Figures 4C,F).

Figures 4C,E illustrate the dependence on prodrug activation rate constant for HAP of Class II (low $K_{O_{2}}$ + bystander) and Class I (high $K_{O_{2}}$, no bystander) respectively. It should be noted that in the no-bystander simulations the effector potency, *a*, was set to a ~fivefold lower value (0.0414 μM^−1^) than for Class II in Table 2. This was done in order to achieve similar overall (average) killing additional to radiation in the tumor microregion for Class I and II HAP under the default conditions (shown in black). Under these conditions, the Class I HAP provides more killing at intermediate O_2_ concentrations (~0.2–10 μM), while the Class II HAP provides much higher cell kill under severe hypoxia (<0.2 μM O_2_).

**Table 2:** **Parameters and variables of the SR-PK/PD model (base model)**.

Parameter	Value	Unit	Description
AUC_*P* inflow_	50.0	μM/h	Unbound AUC of prodrug in inflowing vessels
*k*_met *P*,max_	0.01	s^−1^	Maximum rate constant for metabolism of prodrug to effector (under anoxic conditions)
$K_{O_{2}}$	0.126 (low); 1.26 (high)	μM; μM	O_2_ concentration for half-maximum prodrug activation, assumed to be equal to the O_2_ concentration for half-maximum cytotoxicity of PR-104A (low $K_{O_{2}}$) and tirapazamine (high $K_{O_{2}}$) determined in SiHa single cell suspensions (31)
*k*_metE_	0.01	s^−1^	Rate constant for loss of effector
*K*_t_*_P_*	0.1	s^−1^	Rate constants for transfer from the extracellular to the intracellular compartment (*k*_t_*_N_*) for prodrug and effector
*K*_t_*_E_*	0.01	s^−1^	
*D*_P_	10^−6^	cm^2^/s	Tissue diffusion coefficients for prodrug and effector. Reported values are the volume-averaged parameters of the extracellular diffusion coefficients *D_eN_* with: *D* = φ_e_ *De_N_*
*D*_E_	10^−6^	cm^2^/s	
φ_i_	0.4		Intracellular volume fraction in tumors
*A*	0.200	μM^−1^h^−1^	Proportionality constant for the PK/PD model for +bystander case
	0.0414	μM^−1^h^−1^	Proportionality constant for no-bystander case
α_H_	0.0663	Gy^−1^	Proportionality constants for the linear-quadratic model for radiosensitivity under hypoxia
β_H_	0.0028	Gy^−2^	
OER_α_ = OER_β_	2.8		Maximal O_2_ enhancement ratios for the α and β components of the linear-quadratic model (91)
*K_ms_*	4.2	μM	O_2_ concentration for half-maximum radiosensitivity calculated from Ref. (91)
*D*_r_	15	Gy	Radiation dose

*The parameter and variables are similar to those estimated for our previous SR-PK/PD model for PR-104 in SiHa tumors (35)*.

**Figure 5:**
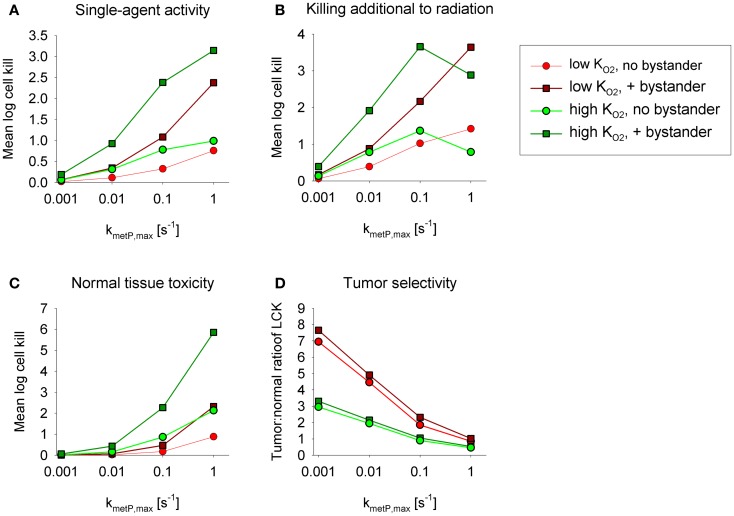
**Impact of the prodrug activation rate constant, *k*_met*P*,max_, on overall cell killing in the tumor microregion of low-K_O2_ (red) and high-K_O2_ (green) HAP with and without bystander effects**. Average log cell kill in the tumor microregion by HAP without radiation **(A)** and additional to radiation **(B)**. The latter was calculated as the difference between overall log cell kill by prodrug + radiation and log cell kill by radiation alone. **(C)** Average HAP-mediated killing in a hypothetical normal tissue in which cells are assumed to have the same intrinsic sensitivity as tumor cells, based on simulations in a microvascular network of a mapped rat cremaster muscle region. **(D)** Tumor:normal tissue ratio of cell killing.

Figures 5A,B show overall HAP-mediated killing, averaged across the whole tumor microregion, for the cases demonstrated in Figure 4. In all cases, monotherapy activity increased with increasing prodrug activation rates over the range examined (*k*_met*P*,max_ = 0.001–1 s^−1^), although more markedly for low-$K_{O_{2}}$ compounds (shown in red). This difference was even more pronounced for killing of radiobiologically hypoxic cells (i.e., killing additional to radiation; Figure 5B), in which case activity of high-$K_{O_{2}}$ compounds (shown in green) decreases at high rates of metabolism reflecting a severe penetration problem (which is not fully offset by allowing bystander diffusion). Class II HAP (dark red) are thus more tolerant of high values of *k*_met*P*,max_ than Class I HAP (light green).

The above analysis suggests that monotherapy activity increases monotonically with *k*_met*P*,max_ up to very high values, but it is important to consider whether this changes selectivity relative to normal tissues. To address this we used the above cremaster muscle microregion (Figure 3), to represent a generic well-perfused normal tissue with mild physiological hypoxia. For the present purpose, we assumed that this “normal tissue” is populated by cells with the same intrinsic sensitivity to effector as cells in the tumor microregion. Cell kill in the normal tissue region showed an even steeper dependence on *k*_met*P*,max_ than the tumor region (Figure 5C). As a consequence, tumor selectivity (assessed using the tumor:normal tissue ratio of log cell kill) fell as rates of bioreductive metabolism increased (Figure 5D). This falling therapeutic ratio reflects the greater compromise to delivery of rapidly metabolized prodrugs in the tumor network because of lower blood flow rates, longer diffusion distances, and more severe hypoxia than in the normal tissue (Figure 3). The model confirmed our expectation that toxicity to physiologically hypoxic normal tissues will be higher for a high-$K_{O_{2}}$ HAP (Figure 5C).

### Modulation of prodrug diffusion properties

**Figure 6:**
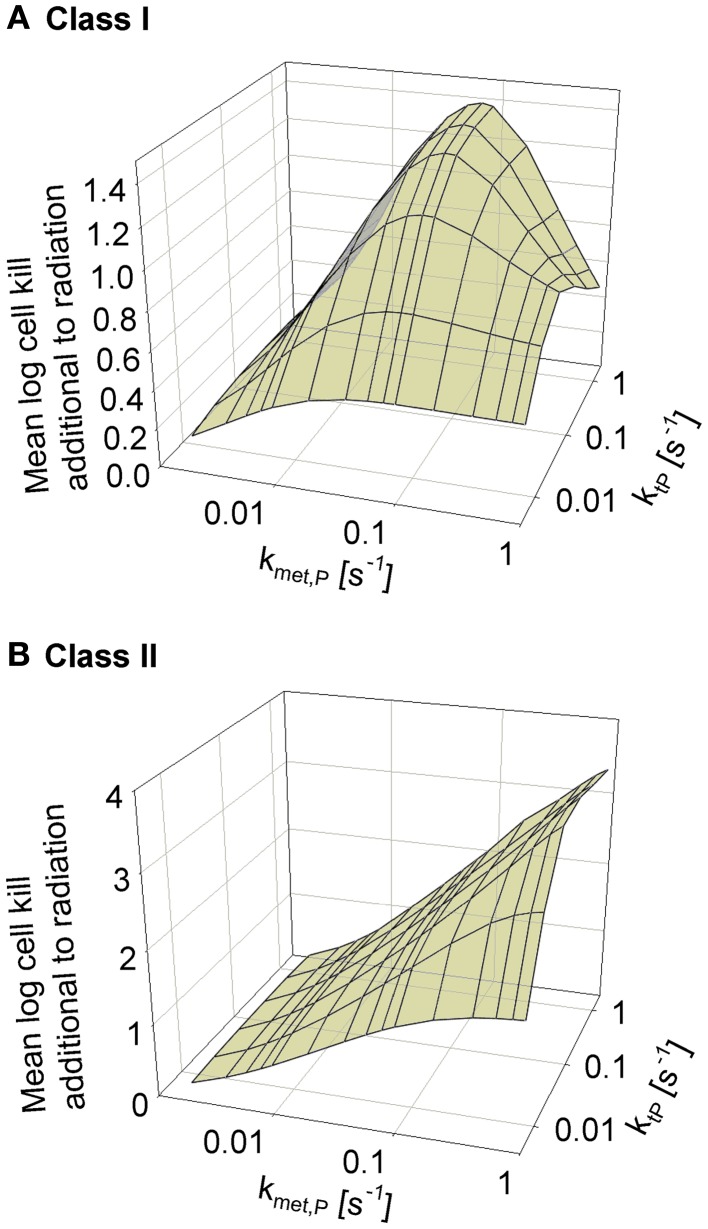
**Dependence of HAP-induced cell killing additional to radiation on *k*_met*P*,max_ and the membrane transfer rate constant *k*_t_*_P_***. **(A)** Class I HAP; **(B)** Class II HAP. Simulations for Class I HAP (no-bystander) were performed as described in the legend of Figure 4.

We next asked whether the activity of class II HAP is sensitive to the diffusibility of the prodrug, as previously shown by the SR-PK/PD model for tirapazamine analogs (54). In the latter study, tissue diffusion was defined by a single parameter, the effective diffusion coefficient as measured by flux through multicellular layer cultures; this represents the weighted averages of the free drug diffusion coefficient within cells, across membranes and in the extracellular matrix, based on treating tissue as homogenous space. In contrast, the present model explicitly considers extra- and intracellular compartments, and overall diffusion is determined by two parameters: the rate constant for transfer between extra- and intracellular compartments, *k*_t_*_P_*, and the diffusion coefficient in the extracellular compartment *D*_P_ (Figure 2). Since changes in the physicochemical properties of HAP are expected to affect membrane permeability more than paracellular diffusion, *k*_t_*_P_* was modulated. The results (Figure 6) show that hypoxic cell killing for Class I HAP is optimal at *k*_met*P*,max_ ~0.1 s^−1^ [Figure 6A; consistent with the previous SR-PK/PD model for tirapazamine (54)]. Class I HAP also showed an optimum value of *k*_t_*_P_*, reflecting the fact that the membrane transfer rate constant controls how much prodrug is available for intracellular activation. In contrast, for Class II HAP killing additional to radiation increased monotonically over the full range of *k*_met*P*,max_ and *k*_t_*_P_* (Figure 6B) reflecting the tolerance of high rates of intracellular prodrug activation owing to a low $K_{O_{2}}$ and because effector diffusion offsets compromised prodrug penetration.

### Modulation of the effector diffusion range

**Figure 7:**
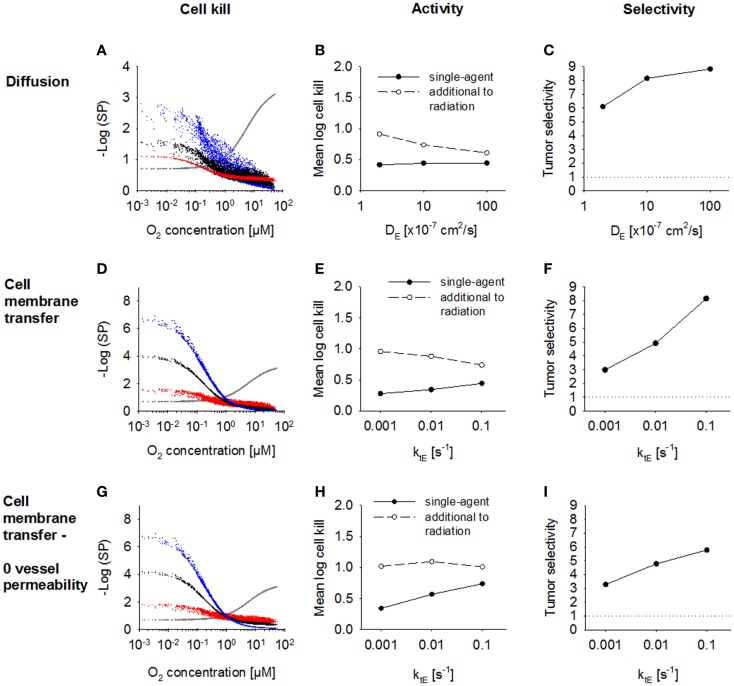
**Modulation of effector diffusion properties for a class II HAP**. SR-PK/PD model simulations using the base model (Table 2), with modulation of the following effector parameters: **(A–C)** the extracellular diffusion coefficient *D_E_*, using a 10-fold higher value for the membrane transfer rate constant *k*_t_*_E_*; **(D–I)** membrane transfer rate constant *k*_t_*_E_*, with **(D–F)** or without **(G–I)** blood vessel permeability to the effector. Left panels show survival probability (as a function of O_2_ in the FaDu tumor microregion) after 15 Gy radiation (gray) or HAP (black: base model; red and blue: 10-fold higher and lower parameter value, respectively). Middle panels show average monotherapy activity (black, solid lines) and killing additional to radiation (white, dashed lines) in the tumor region. Right panels show the tumor:normal tissue ratio of cell killing.

Next, we investigated the dependence of antitumor activity on effector parameters for Class II HAP. Since tissue transport of the effector is dependent on its membrane transfer (*k*_t_*_E_*), paracellular diffusion (*D_E_*) and its stability (*k*_met_*_E_*), these parameters were modulated ~10-fold relative to the base model. In order to see an impact of a change in the paracellular diffusion coefficient *D_E_, k*_t_*_E_* had to be increased 10-fold to 0.1s^−1^, indicating that effector transport is otherwise limited by membrane transfer. An increase in diffusion coefficient, *D_E_* (Figure 7A) caused increased diffusion of the effector away from the hypoxic regions where it is formed, which decreased killing in hypoxic regions and increased killing in aerobic regions. As a consequence, overall killing in addition to radiation decreased and monotherapy activity increased (Figures 7B,C). A similar effect could be observed when increasing the membrane transfer rate constants *k*_t_*_E_* (Figures 7D,E). However, the effect of increased diffusibility (higher *D_E_* or *k*_t_*_E_*) on single-agent activity was only minor because the effector was defined to freely permeate the blood vessel walls, and thus lost into the vasculature. The protective effect of this washout was confirmed by simulations with zero vessel permeability to the effector, which showed a much larger increase in HAP monotherapy activity with increasing *k*_t_*_E_* (Figures 7G,H). Notably, an increase in effector diffusibility in the presence of vessel permeability elevated the tumor:normal tissue ratio of killing (Figures 7C,F), reflecting that washout of effector into the circulation is more protective in normal tissue (with lower distances to nearest vessel; Figure 3) than in tumor tissue.

**Figure 8:**
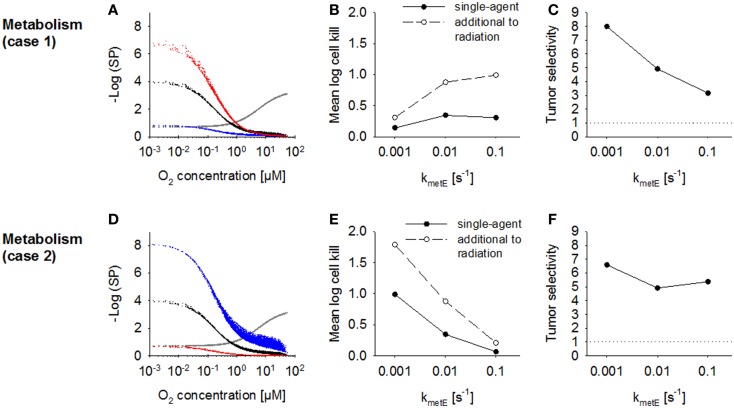
**Modulation of effector stability for a class II HAP**. SR-PK/PD model simulations using the base model (Table 2), or modulation of the rate constant for loss of effector *k*_met_*_E_*. **(A–C)** Case 1 where survival probability is proportional to reactivity (*k*_met_*_E_*); **(D–F)** Case 2 where survival probability is independent of *k*_met_*_E_*. See legend of Figure 7 for description of graphs.

The effect of increasing the rate constant for effector reaction *k*_met_*_E_* was investigated using the default case where cell killing scales with effector reactivity (case 1) and alternatively where cell killing is independent of reactivity (case 2). The proportionality constant *a* was set to the values given in Table 2 to achieve equivalent killing of the two effector types at the default *k*_met_*_E_* of 0.01 s^−1^. In case 1, an increase in *k*_met_*_E_* produces a proportional increase in the rate of formation of cytotoxic lesions and resulted in higher killing in hypoxic regions (Figure 8A). Killing in aerobic regions (>4 μM O_2_) was decreased at high values of *k*_met_*_E_* due to associated low effector penetration, and this limited overall activity (Figure 8B). Tumor selectivity decreased with increasing *k*_met_*_E_* (Figure 8C) due largely to increasing cell killing in normal tissue.

In case 2 (where cytotoxic lesion production is independent of effector reactivity) decreasing *k*_met_*_E_* resulted in higher killing across the whole tumor microregion (Figure 8D), with a large increase in HAP activity, both as a single agent and in addition to radiation, with increasing effector stability (Figure 8E). Killing in the normal tissue microregion was affected to a similar extent, so that tumor selectivity was comparable for different values of *k*_met_*_E_* (Figure 8F).

### Estimation of the impact of effector washout by blood flow

**Figure 9:**
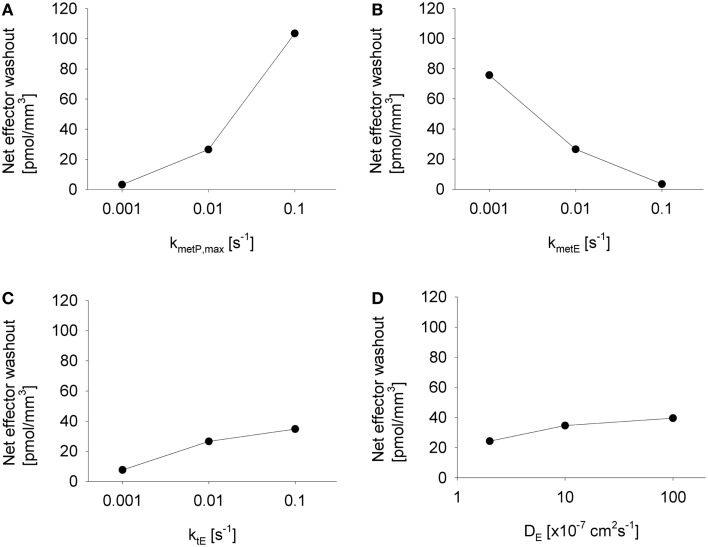
**Impact of different parameter modulations on effector washout**. Graphs show the net transport of effector across the vessel walls in the FaDu tumor microregion, normalized to the volume of the region, as estimated by the SR-PK/PD model with modulation of **(A)** *k*_met*P*,max_, **(B)** *k*_met_*_E_*, **(C)** *k*_t_*_E_*, and **(D)** *D_E_*, with unmodulated parameters held at their default values from Table 2, except in **(D)** where 10-fold higher values for *k*_t_*_E_* was used.

The above analysis demonstrated that washout of effector by blood flow can, under some conditions, significantly protect perivascular cells from cytotoxicity. This raises the question whether effector washout might have pharmacodynamic effects in downstream tumor regions (redistribution of effector through the tumor vasculature), or might result in significant systemic exposure to the effector. To test the sensitivity of effector washout on different parameters, we used the Class II HAP model to calculate net transport of effector across the tumor/plasma boundary in each vessel segment of the tumor microregion. This showed high sensitivity to the rate constant of effector production (*k*_met*P*,max_) and effector stability (*k*_met_*_E_*) but only moderate sensitivity to effector diffusibility (*k*_t_*_E_* and *D_E_*) (Figure 9), because the former parameters substantially affect overall effector concentrations.

**Figure 10:**
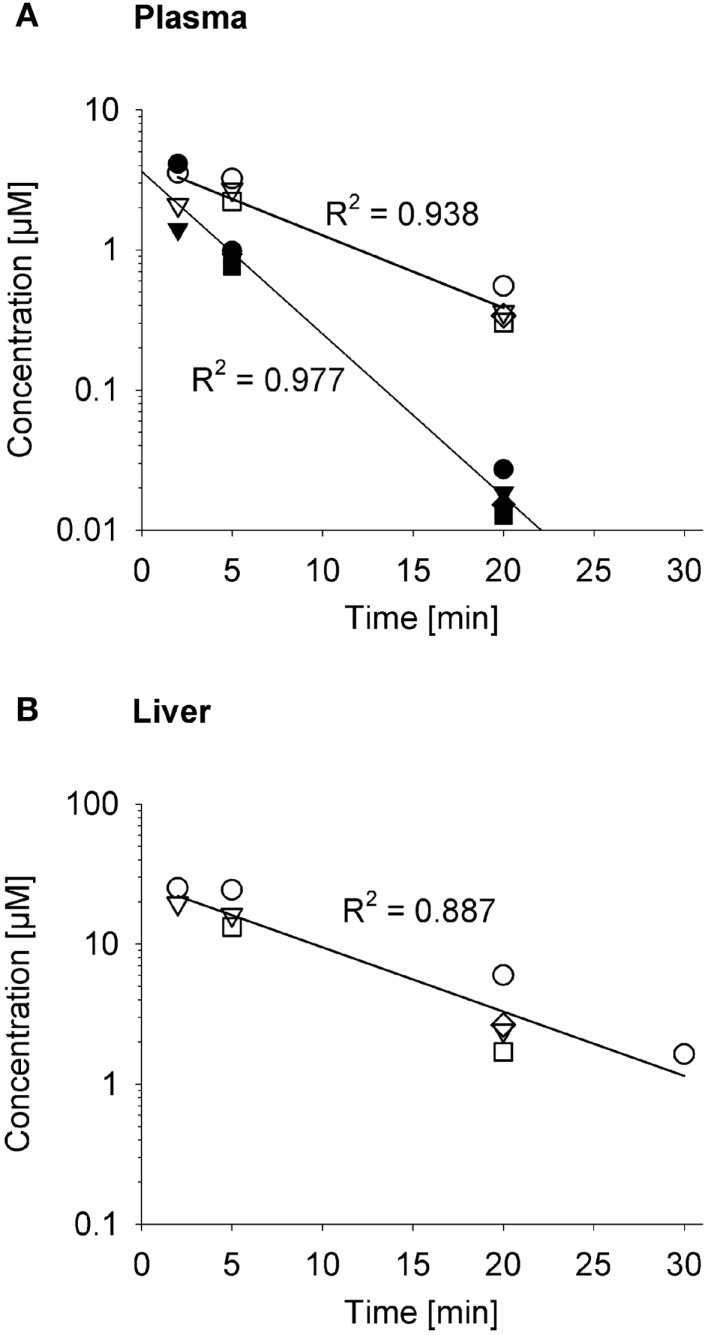
**Pharmacokinetics of PR-104H and its reduction product PR-104M in NIH-III nude mice**. Concentrations of PR-104H (filled symbols) and PR-104M (open symbols) in plasma **(A)** and liver **(B)** following i.v. administration of 10 μmol/kg PR-104H, with linear regression lines. At each time point different symbols represent data from individual mice.

The impact on systemic exposure will depend on the distribution volume (*V*_D_) and clearance (CL) of the effector. To evaluate a specific case, we returned to the SR-PK/PD model for PR-104 (35) because the plasma AUC of its active metabolites (PR-104H and PR-104M) after dosing PR-104 has been determined in three strains of mice (35, 52, 61). To facilitate this analysis, we measured the pharmacokinetics of PR-104H and PR-104M in plasma and liver following i.v. dosing of NIH-III nude mice with synthetic PR-104H (Figure 10). This showed a very short half-life (2.6 min) for PR-104H in plasma, and high PR-104M concentrations in plasma and liver, indicating rapid conversion of PR-104H to PR-104M. The half-lives of PR-104M in plasma and liver (~7 min) were similar to the half-life of PR-104M in culture medium at 37°C [6.6 min (35)], suggesting that CL of PR-104M is mainly due to its chemical instability.

**Figure 11:**
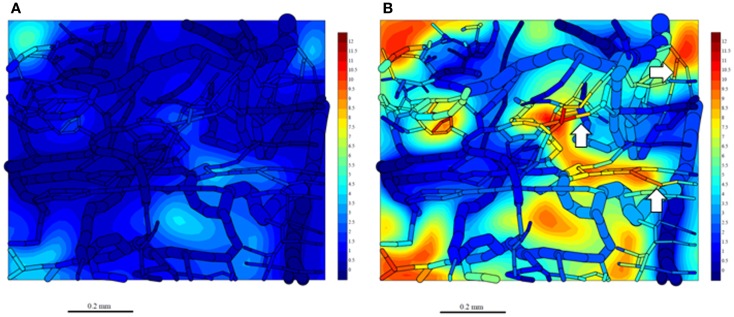
**Simulation of PR-104M, the second active metabolite of PR-104A, in the tumor microregion**. PR-104M AUC was calculated in the FaDu tumor microregion with oxygenation as shown in Figures 3A,F, using our previously published PR-104 SR-PK/PD model (35) at a PR-104 dose of 562 μmol/kg with inflow AUC of PR-104H and PR-104M set to 0 to distinguish the impact of reduced metabolites formed in the tumor. **(A)** Simulation for an HCT116/WT tumor. **(B)** Simulation for a HCT116/sPOR#6 tumor (20× higher prodrug activation rate constant). The AUC (in micromoles hour) is shown in color scale in the whole FaDu microvascular network projected into one plane, and in a mid-plane section of the microregion (as a contour plot). White arrows indicate vessels with high concentrations of active metabolite.

Since washout was most sensitive to *k*_met*P*,max_ (Figure 9), we performed PR-104 SR-PK/PD model simulations using two different values of this parameter, corresponding to rates measured *in vitro* for HCT116/WT and a cell line with 20-fold higher *k*_met*P*,max_ (HCT116/sPOR#6) (35). This confirmed that washout of metabolites produced in hypoxic regions can increase plasma levels in nearby vessels, an effect that was more pronounced at the higher *k*_met*P*,max_ (white arrows in Figure 11). Using *V*_D_ and CL estimates from measured plasma PK following administration of PR-104 or PR-104H (Table 3), we calculated that washout would elevate the free plasma AUC of PR-104H+M in NIH-III nude mice [estimated from the measured PK after i.p. administration of 562 μmol/kg PR-104 (35)] by only 0.8% in case of HCT116/WT tumors, and 3.3% in case of HCT116/sPOR#6 tumors (Table 3). This was calculated assuming all PR-104H+M was released at once and hence represents a maximum addition to the circulating metabolites. This is consistent with published data showing similar plasma concentrations of reduced metabolites in NIH-III nude mice with HCT116/WT and HCT116/sPOR#6 tumors 30 min after i.p. administration of 562 μmol/kg PR-104 (35).

**Table 3:** **SR-PK/PD model estimation of the free AUC of PR-104A, PR-104H, and PR-104M extracted from or released into plasma by a HCT116 tumor following i.p. administration of 562 μmol/kg PR-104**.

	Tumor	PR-104A	PR-104H	PR-104M
Net amount extracted (−)/released (+) in the tumor microregion (pmol/mm^3^)	WT	−80.2	3.37	11.77
	sPOR#6	−300	14.4	48.7
Net amount extracted (−)/released (+) by a 600 mm^3^ tumor in 1 h (nmol)	WT	−48.1	2.02	7.06
	sPOR#6	−180	8.61	29.2
*V*_D_ (l/kg)	–	2.6^a^	2.8^b^	2.8^b^^,^^c^
CL (l/h/kg)		5.0^d^	44^b^	20^b^^,^^c^
Free AUC extracted (−)/released (+) by a 600 mm^3^ tumor (μM.h)	WT	−0.286	0.00179	0.0140
	sPOR#6	−1.07	0.00759	0.0578
Free AUC in plasma (μM.h)^c^	–	61.5	1.30	0.71

*Using our previous PR-104 SR-PK/PD model for HCT116/WT tumors and HCT116/sPOR#6 tumors [with a 20-fold higher prodrug activation rate constant (35)], simulations were performed for a PR-104 dose of 562 μmol/kg, with inflow AUC of PR-104H and PR-104M from extra-tumor sources set to 0 to identify the flux of reduced metabolites formed in the tumor. Net transport of PR-104A, PR-104H, and PR-104M across the tumor-plasma boundary in the tumor microregion was calculated, scaled to the volume of a typical HCT116 tumor (600 mm^3^), and divided by the clearance (CL) for a 25 g mouse to calculate the AUC reached in plasma. The distribution volume (*V*_D_) and CL were determined in pharmacokinetic studies (see footnotes)*.

*^a^Estimated for CD-1 mice following i.v. administration of 56.2 μmol/kg PR-104A (92)*.

*^b^Estimated from the plasma PK of PR-104H and PR-104M measured in NIH-III nude mice after i.v. administration of 10 μmol/kg PR-104H (Figure 10)*.

*^c^Assuming that *V*_D_ of PR-104M is equal to *V*_D_ of PR-104H*.

*^d^Estimated by non-compartmental analysis of the PR-104A plasma PK measured in NIH-III nude mice after i.p. administration of 562 μmol/kg PR-104 (35)*.

It should be noted that all simulations in this study were performed at blood flows where prodrug delivery and effector extraction were not highly perfusion limited. To demonstrate this (and the effect of blood flow on increasing effector washout into the systemic circulation) we increased the blood flow rate, *Q*, 2.5-fold, while decreasing inflow *p*O_2_, to maintain a similar hypoxic fraction (Figures S1A,B in Supplementary Material). Under conditions of high prodrug metabolism (*k*_met*P*,max_ of 1 s^−1^), this increased prodrug extraction from the plasma 1.5-fold and effector washout into the plasma twofold (Figure S1C in Supplementary Material) but overall killing in the microregion increased by only 26% (Figures S1D,E in Supplementary Material) reflecting little increase in perivascular effector concentrations.

## Discussion

In this study, SR-PK/PD modeling was utilized to identify strategies for optimization of HAP. The model has a solid biological foundation because it is based on our previous SR-PK/PD model for PR-104 for which parameters have been determined experimentally using three cell lines (35). The latter model was the first to explicitly consider the intra-tumor distribution of bystander metabolites.

### Normal tissue toxicity

A novel feature of the present study is the comparison of predictions for a tumor and normal tissue network, the latter based on a mapped microvascular network in a rat cremaster muscle (56) with oxygen consumption rate adjusted to simulate mild hypoxia (Figure 3). This comparison is important because the utility of HAP is likely to be constrained by the requirement that prodrug activation at intermediate O_2_ does not cause toxicity in tissues with physiological hypoxia, such as liver (43–45), retina (66), and possibly bone marrow (49). The low tumor:normal tissue ratios of killing determined in our study (Figures 5, 7, and 8) point to limitations on achieving tumor selectivity with HAP. However, it is important to note that our model makes the assumption that intrinsic cellular sensitivity to the released effector (potency parameter *a*) is the same for cells in the tumor and normal tissue networks. Most effectors of HAP in clinical development are DNA-reactive cytotoxins (13) that provide some tumor-specificity due to the cytokinetics and DNA repair abnormalities (67) of tumor cells relative to normal cells. In addition, release of HAP effectors that target oncogenic pathways in tumor cells has potential for conferring additional tumor selectivity (68). Normal tissue models will need to be revisited when more quantitative information about oxygenation and drug targets in potentially dose-limiting normal tissues is available. Nevertheless, the present results emphasize the importance of using HAP strategies to augment selectivity of effectors that already provide some level of tumor selectivity, rather than as the sole mechanism of tumor targeting.

In addition, normal tissue toxicity due to washout of effectors has been a concern in the development of targeted anticancer prodrugs (69, 70) although to our knowledge no clear link between toxicity and increased effector levels has been established. In the current study systemic toxicity of HAP as a result of release of active metabolites from the tumor appears unlikely; our model suggests that a significant contribution to toxicity would require a combination of extreme assumptions (high tumor burden, intra-tumor activation rates, effector stability, tumor blood flow rates, as well as low systemic CL of effector). However, toxicity in normal tissue sharing a microcirculatory system with the tumor remains a possibility.

### Optimal prodrug parameters of class I and class II HAP

This study compared an idealized Class I HAP (high $K_{O_{2}}$ with no-bystander effect, illustrated by tirapazamine and SN30000) and a Class II HAP (low $K_{O_{2}}$ with bystander effect, e.g., PR-104A and TH-302) to assess which of these HAP strategies is better suited for complementation of radiotherapy. Our previous tirapazamine SR-PK/PD model showed that Class I HAP exhibit an optimum *k*_met*P*,max_, above which hypoxic cell killing decreases because increasing consumption of the prodrug limits its penetration to hypoxic regions (54). This finding could be replicated using the present model, in which intra- and extracellular compartments are made explicit (Figure 5). In contrast, activity of Class II HAP was predicted to increase monotonically with increasing *k*_met*P*,max_ in the investigated range of 0.001–1 s^−1^; we consider this range to be physiologically and pharmacologically relevant based on measured rate constants (32, 35, 54, 60). In addition, the potential to further increase *k*_met*P*,max_ by drug design would be limited by membrane transport and by the concurrent decrease in tumor selectivity (Figure 5D).

The superiority of Class II over Class I HAP at high *k*_met*P*,max_ can be explained by two factors. Firstly, the lower $K_{O_{2}}$ value minimizes metabolic loss during diffusion into hypoxic target regions (compare Figures 4A,D). Secondly, bystander effects alleviate the impact of poor prodrug penetration due to redistribution of effector between regions of different prodrug exposure. Both of these factors also explain the lower sensitivity of antitumor activity to prodrug diffusibility of class II relative to class I HAP (Figure 6).

### Design of optimized class II HAP

Spatially resolved pharmacokinetic/pharmacodynamic modeling represents a valuable tool for the rational design of improved HAP, as has been demonstrated by our previous SR-PK/PD model for Class I HAP (54). The insight that there is an optimum for combinations of *D_P_* and *k*_met_*_P_* that can accommodate the competing requirements of efficient prodrug activation and tissue penetration prompted the SR-PK/PD model-guided screening of tirapazamine analogs (71) that identified SN30000 as a prodrug with superior tissue penetration and antitumor activity in combination with radiation (32). The model-based design of class II HAP presents a greater challenge as it requires the specific consideration of the transport parameters of the effector in addition to those of the prodrug. In this study SR-PK/PD modeling was used to identify reaction-diffusion parameters of Class II HAPs that could be optimized. The outcome is summarized in Table 4, which shows that antitumor activity of class II HAP could be enhanced by increasing *k*_met*P*,max_, although this would need to be balanced against the need to maintain sufficient tumor selectivity.

**Table 4:** **Summary of SR-PK/PD modeling results for Class II HAP**.

	Antitumor activity^a^		Tumor selectivity^b^	Systemic effector release^c^
	HAP only	HAP+ radiation		
*k*_met*P*,max_ ↑	↑ ↑	↑ ↑	↓	↑ ↑
*k*_met_*_E_* ↑(case 1)	↑ ↑	↑ ↑	↓	↓ ↓
*k*_met_*_E_* ↓(case 2)	↑ ↑	↑ ↑	~	↑ ↑
*D_E_* ↑	↑	↓	↑	↑
*k*_t_*_E_* ↑	↑	↓	↑ ↑	↑

*↑ increase; ↓ decrease; ~ no substantial change*.

*^a^Overall cell kill predicted in the 3D FaDu tumor microregion (see Figures 5 and 7)*.

*^b^Ratio of overall cell killing in the tumor and cremaster muscle microregions (see Figures 5 and 7)*.

*^c^Overall effector released into the circulation by the FaDu tumor microregion (see Figure 8)*.

When looking at the impact of a change in effector metabolism (*k*_met_*_E_*) we distinguished two types of effectors: an effector that needs to react irreversibly with its target to elicit cytotoxicity, meaning that cell killing is a function of this chemical reactivity as for alkylating agents (Case 1), and an inhibitor where chemical transformation of the effector (whether spontaneously or by metabolism) gives rise to non-toxic products (Case 2). Modeling showed that effector reactivity should be increased in Case I but decreased in Case 2, in order to improve antitumor activity. In Case 1 the increase in overall killing when increasing reactivity by drug design needs to be balanced against a predicted decrease in tumor selectivity.

The model revealed that bystander effects are limited not only by diffusion and reaction of the effector, but also by effector washout, unless this washout targets downstream regions via a blood-borne bystander effect. The blood vessels act as a sink for effector, a problem that has previously been flagged in the context of drug diffusion into peritoneal tumors from the peritoneal cavity (72). Therefore, the choice of optimal effector diffusibility (defined by *k*_t_*_E_* and *D_E_*) depends on the therapeutic context, with low diffusibility generally better for combination with radiation, and high diffusibility preferred for monotherapy activity (Figures 7B,E).

Notably, a greater increase of bystander killing with increasing effector diffusibility has previously been suggested by the finding that bystander effects of dinitrobenzamide mustard prodrugs in multicellular layer co-cultures of NfsB-overexpressing and WT cells correlated with effector lipophilicity (73, 74). This may partially be due to the intimate mixture of activator and target cells in these co-cultures, so that effector washout at the multicellular layer boundaries affects both cell types to the same extent. In contrast, blood vessels in the virtual tumor microregion that act as a sink for effector are generally more distant from hypoxic prodrug-activating regions than from bystander target zones. Therefore, multicellular layer co-cultures have may limited use in assessing antitumor activity for HAP, although they are useful to rank HAP according to the diffusion range of their effectors.

Although an increase in the effector diffusion range provided only a moderate increase in single-agent activity it was found to have a surprisingly large effect in protecting cells in normal tissues where the HAP is activated (Figures 7C,F). Increasing effector diffusibility and prodrug activation rate simultaneously (the latter to compensate for washout) may therefore be a good strategy to improve monotherapy activity.

The suggested HAP model parameters could be modulated in drug design by changing the physicochemical properties that determined these parameters; e.g., one-electron reduction potential in controlling *k*_met*P*,max_ (9, 75, 76) or lipophilicity, pK_a_, molecular weight, and number of H-bond donors and acceptors in controlling diffusibility (77). In most cases any change will affect several model parameters, but the value of the SR-PK/PD model lies in part in its ability to accommodate all such changes.

The high prodrug diffusibility and low effector diffusibility that would be ideal for combination with radiotherapy according to the model predictions may not be achievable in all chemical classes of HAP. In case of dinitrobenzamide mustards (PR-104 analogs), the physicochemical properties of prodrug and effector (other than mustard reactivity) will be similar since their structures only differ in one aromatic substituent. The use of fragmenting prodrugs of aliphatic mustards such as TH-302 relaxes this constraint, providing a higher scope for independent optimization of prodrug and effector diffusion properties.

### Limitations of the model

The present SR-PK/PD model has two key limitations. Firstly, the model uses a small tumor region (0.12 mm^3^) that cannot be expected to fully capture the heterogeneity in vascular density and perfusion in tumors (78). This heterogeneity results in macroregional variations in oxygenation and prodrug delivery that may decrease the overall relevance of bystander effects of Class II HAP. For example, macroscopic, well-perfused, and oxygenated areas [observed in some tumor xenografts (35, 79, 80)] would be beyond the reach of the hypoxia-driven bystander effects studied here, unless there is redistribution of diffusible effectors between differently oxygenated tumor regions via the bloodstream (blood-borne bystander effect). Mapped tumor regions of a larger scale are not yet available but could be used to investigate these macroregional effects in the future. Secondly, our steady-state model does not account for time-dependent processes such as cycling hypoxia (81–83) and the kinetics of reoxygenation, cell proliferation, and cell death. Cycling hypoxia is thought to arise from variations in blood flow (84–86), which would affect not only delivery of oxygen but also that of the HAP. Since cycling hypoxia has a dominant periodicity of two to three cycles per hour (82), it may be relevant even for HAP with a residence time in the circulation of few hours and critically important for HAP with long residence times in tumors as is reoxygenation for effectors with long residence times such as AQ4N (87). The implications of variable blood flow and cycling hypoxia should be incorporated in future SR-PK/PD models.

## Conclusion

In spite of its limitations, the SR-PK/PD model has identified strategies for optimization of HAP worthy of further investigation. This is the first modeling study that attempts to systematically compare the activity of Class I and Class II HAP and the first that integrates the use of a normal tissue network to assess tumor selectivity. It suggests that class II HAP offer the following advantages relative to class I HAP: they tolerate a wider range of *k*_met*P*,max_ (allowing a wider scope for prodrug design as well as their use in a range of human tumors with different one-electron reductase expression), are less sensitive to prodrug diffusibility (thus making it less critical to optimize this parameter) and have lower toxicity in normal tissues with physiological hypoxia (Figures 4C,F). A possible disadvantage is that complementation of radiation falls off rapidly with increasing effector diffusion away from the hypoxic activating region (Figure 7).

The modeling also showed that the design of optimized Class II HAP is complex due to the need to consider many different processes such as metabolism, membrane transfer, paracellular diffusion, and washout. The spatial and temporal heterogeneity of O_2_ and blood flow within tumors (and potentially variations in reductase expression and other determinants of sensitivity) adds further complexity. Therefore it is difficult to design one HAP that fits all tumor types and therapeutic settings. However, important tendencies have been identified by the model, e.g., that antitumor activity may be increased by optimizing effector stability and prodrug activation rates while tumor selectivity may be improved by increasing effector diffusibility.

The present study has implications for targeted anticancer prodrug approaches, as most are limited by spatial heterogeneity in prodrug activation (3, 4, 88). The approach is also potentially applicable to hypoxia-targeting strategies that utilize prodrugs such as gene-directed enzyme prodrug therapy approaches in which the prodrug-activating enzyme is delivered using macrophages that accumulate in hypoxic tumor regions (89), or obligate anaerobic bacteria (90). In approaches with a different spatial distribution of prodrug-activating regions relative to non-activating regions and blood vessels the optimum conditions may differ and could be identified in the future using SR-PK/PD modeling.

## Author Contributions

Annika Foehrenbacher, William R. Wilson, and Kevin O. Hicks conceived and designed the study. Timothy W. Secomb developed the Green’s function method and wrote the program to simulate multiple intracellular and extracellular solutes. Annika Foehrenbacher designed and ran the simulations and Annika Foehrenbacher, Kevin O. Hicks, and William R. Wilson analyzed the data. Annika Foehrenbacher performed the PR-104H pharmacokinetic study and assembled the figures, table, and manuscript. Annika Foehrenbacher, Kevin O. Hicks, William R. Wilson, and Timothy W. Secomb wrote the paper.

## Conflict of Interest Statement

The authors declare that the research was conducted in the absence of any commercial or financial relationships that could be construed as a potential conflict of interest.

## Supplementary Material

The Supplementary Material for this article can be found online at http://www.frontiersin.org/Journal/10.3389/fonc.2013.00314/abstract

Figure S1 **Impact of blood flow rate on tumor PK/PD of HAP**. SR-PK/PD model simulations were performed using the default O_2_ transport parameters in Table 1 (*Q* = 40 nl/min; *p*O_2_ = 40 mm Hg; black) and using a higher blood flow rate and lower inflow *p*O_2_ (*Q* = 100 nl/min; *p*O_2_ = 29 mm Hg; red). The prodrug activation rate constant *k*_met*P*,max_ was set to a high value of 1 s^−1^ with all other parameters as in Table 2. **(A)** O_2_ concentration as a function of distance to nearest vessel in the FaDu tumor microregion. **(B)** The resulting oxygen distribution for the higher blood flow rate case (c.f. Figure 3F). **(C)** Net amount of prodrug and effector extracted from plasma (− sign) or released into plasma (+ sign). **(D)** Intracellular prodrug concentration *C_iP_* as a function of O_2_ in the FaDu tumor microregion. **(E)** Resulting killing. Lines indicate average killing in the tumor region (2.47 and 3.04 logs of cell kill for low flow and high flow simulations respectively).
