# Supplementary figures and images for: Design of Optimized Hypoxia-Activated Prodrugs Using Pharmacokinetic/Pharmacodynamic Modeling
Source: Front Oncol. 2013 Dec 27;3:314. doi: 10.3389/fonc.2013.00314 (PMC3873531; doi:10.3389/fonc.2013.00314)

**A**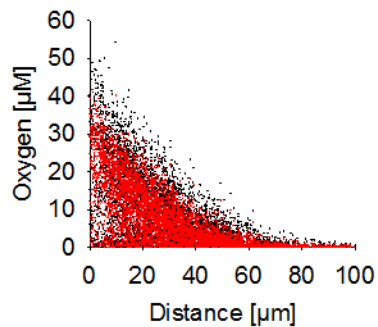**B**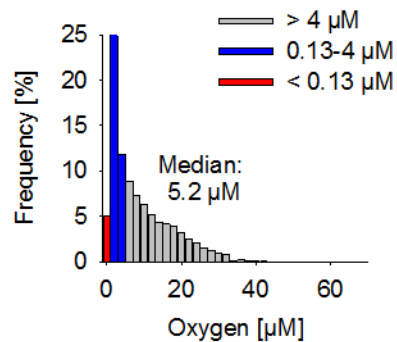**C**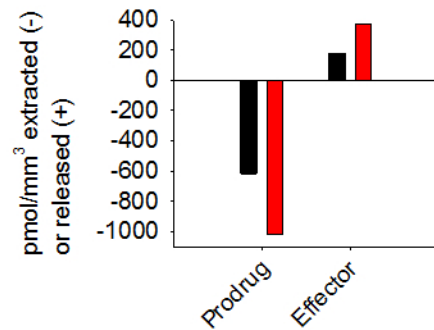**D**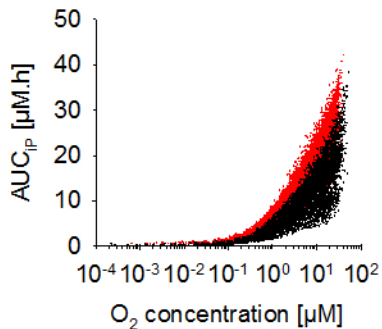**E**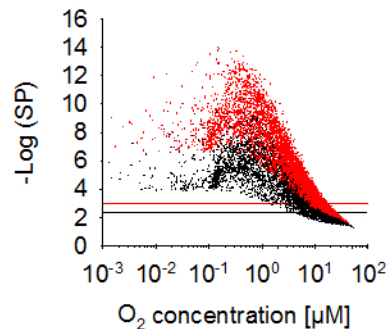

Supplement: Figure S1 — Impact of blood flow rate on tumor PK/PD of HAP. SR-PK/PD model simulations were performed using the default O2 transport parameters in Table 1 (Q = 40 nl/min; pO2 = 40 mm Hg; black) and using a higher blood flow rate and lower inflow pO2 (Q = 100 nl/min; pO2 = 29 mm Hg; red). The prodrug activation rate constant kmetP,max was set to a high value of 1 s−1 with all other parameters as in Table 2. (A) O2 concentration as a function of distance to nearest vessel in the FaDu tumor microregion. (B) The resulting oxygen distribution for the higher blood flow rate case (c.f. Figure 3F). (C) Net amount of prodrug and effector extracted from plasma (− sign) or released into plasma (+ sign). (D) Intracellular prodrug concentration CiP as a function of O2 in the FaDu tumor microregion. (E) Resulting killing. Lines indicate average killing in the tumor region (2.47 and 3.04 logs of cell kill for low flow and high flow simulations respectively). [file 74594_Hicks_Presentation1.PDF]
